# Recent Developments in Eco-Friendly Wood-Based Composites II

**DOI:** 10.3390/polym15081941

**Published:** 2023-04-19

**Authors:** Pavlo Bekhta

**Affiliations:** 1 Department of Wood-Based Composites, Cellulose and Paper, Ukrainian National Forestry University, 790 57 Lviv, Ukraine; bekhta@nltu.edu.ua; 2 Department of Wood Science and Technology, Faculty of Forestry and Wood Technology, Mendel University in Brno, Zemědělská 3, 613 00 Brno, Czech Republic; 3 Department of Furniture and Wood Products, Technical University in Zvolen, T.G. Masaryka 24, 960 01 Zvolen, Slovakia

Traditional wood-based composites are bonded with synthetic formaldehyde-based adhesives [1,2]. These adhesives bring certain environmental problems because they release formaldehyde emissions, which are a human carcinogen and toxic for the environment [3]. It is difficult to find new uses or new fields for wood-based products because of the lack of proper adhesives which meet the wood industry requirements of being eco-friendly, low-cost, and easy to use. For this reason, growing ecological and environmental consciousness drives efforts for the development of new eco-friendly wood-based composites for various end-use applications. In recent years, significant efforts have been made to reduce formaldehyde emissions from wood-based composites via: (i) the reduction of formaldehyde content in resin formulation [4,5]; (ii) the use of scavengers such as tannins, lignin, starch, wheat and hemp flour, and pulp and paper sludge [6,7,8,9,10,11,12,13,14] or other compounds (starch derivatives, charcoal, pozzolan, zeolites, and urea) [15,16,17,18,19] that scavenge formaldehyde; (iii) the post-treatment or surface treatment of the wood-based products [18,20]; (iv) the use of natural resins, including soy protein, tannin, lignin, and starch adhesives [21,22,23]; (v) and the thermal pre-treatment of veneer before bonding [24,25,26]. Comprehensive information on the reduction of formaldehyde emissions in various ways can be also found in several published reviews [4,20,27,28,29,30]. The most acceptable and effective procedure for reducing formaldehyde emissions in wood-based panels is the use of formaldehyde scavengers, which can be classified as synthetic scavengers, bio-based (natural) scavengers, and nano-scavengers [30].

Identifying additives to reduce the total amount of urea-formaldehyde (UF) resin needed without adversely affecting the panel properties is one way to reduce the negative environmental footprint of UF resins caused by the release of formaldehyde. The results provided by Taghiyari et al. [31] showed that small amounts of micron-scale wollastonite could serve as a resin extender. Sugar palm fiber (SPF) was employed as a reinforcement material in a polyvinyl butyral (PVB) polymer matrix to develop SPF-PVB eco-friendly laminated composites through the hot compression method [32]. The laminated composite sample with 80% of PVB and 20% of SPF showed the highest stiffness value. Thermoplastic starch (TPS) and poly (lactic acid) (PLA) are among the most promising biodegradable polymers that have the potential to replace petroleum-based polymers. The study conducted by Nazrin et al. [33] reveals the potential of PLA/TPS blend bionanocomposites for biodegradable packaging applications. The properties of co-extruded wood/polyethylene composites (Co-WPCs) were improved by filling the shell and wood fiber layers with low-cost nano-silica (nSiO_2_) and micro-silica (mSiO_2_) [34].

One of the possible directions to achieve this goal is the creation of wood composites based on environmentally friendly products, where thermoplastics and their copolymers (low- and high-density polyethylene, polypropylene, co-polyamide, and co-polyester, etc.) are used as adhesives [35,36,37,38,39]. Bark flours obtained from different tree species having a high polyphenol content also exhibited formaldehyde-scavenging properties [40,41,42,43,44,45]. Equally exciting and revolutionary was the development of the use of citric acid (CA) as a green modifying agent and adhesive for wood [2,46]. There is an excellent review [47] whereby the bonding mechanism and types of wood composites bonded with CA are presented. The authors also discussed the best working conditions for the CA in the fabrication of wood composites. The environmental impacts and future outlook of CA-treated wood and bonded composite are also considered. Another alternative to the use of synthetic formaldehyde-based adhesives is to manufacture binderless wood composites [48], since wood is a natural polymer material which is rich in lignocellulosic compounds such as cellulose, hemicellulose, and lignin.

This Special Issue, entitled “Recent Developments in Eco-Friendly Wood-Based Composites II”, comprises 12 high-quality original research and reviews papers by 62 authors from 10 countries on three continents: Asia (China, India, Indonesia, Malaysia), Europe (Austria, Czech Republic, Portugal, Romania, Ukraine), and Africa (South Africa). The papers provide examples of the most recent developments in eco-friendly wood-based composites.

In their paper, Xiao et al. [49] applied a saturated steam heat treatment in a useful way to effectively enhance the dimensional stability and mold-resistance property of bamboo and bamboo-based products. By promoting greenhouse gas sequestration, bamboo and bamboo-based products can improve carbon storage, thereby helping to reduce greenhouses gas emission through replacing traditional products such as concrete, steel, and alloys. The authors observed the decrement of hemicellulose and cellulose after thermal modification, whereby the bamboo samples exhibited improved dimensional stability and anti-fungal properties. The hardness and modulus of elasticity (MOE) of the thermally modified bamboo were 0.75 and 20.6 GPa, respectively.

A very interesting study by Bekhta et al. [50] aimed to evaluate the possibility of using wood particles from deadwood in the production of particleboards. The authors investigated the physical and mechanical properties as well as the formaldehyde content of UF-bonded particleboards with different content of deadwood particles (0%, 25%, 50%, 75%, 100%). It was found that replacing conventional health wood particles with deadwood particles led to the deterioration of the mechanical properties of the boards. In addition, the boards from deadwood particles absorbed more water and swelled more. However, it was shown that adding 3% of MUF resin to UF adhesive increased the bending strength (MOR), MOE, and internal bond strength (IB) by 44.1%, 43.3%, and 294.4%, respectively, while decreasing the water absorption (WA) 24 h and thickness swelling (TS) 24 h by 18.2% and 42.9%, respectively. Moreover, a significant advantage was that boards made from 100% deadwood particles are characterized by 34.5% less formaldehyde content than reference boards made from conventional health wood.

In the study by Ismail et al. [51], a new approach to fabricate the coconut shell nano-biocomposites using waste polypropylene plastic packaging as a matrix was proposed. Coconut shell, an agricultural waste, was bonded with waste plastic to form a biocomposite with a coupling agent. The authors investigated the optimum percentage composition and the effect of coconut shell ball milling time on the physical, mechanical, and thermal properties of the biocomposite. They found that the properties of the biocomposite could be improved by reducing the particle size of the coconut shell (increasing the duration of milling). As the milling time increased from 0 to 40 h, the density increased from 0.9 to 1.02 g/cm^3^; TS decreased from 3.4 to 1.8%; porosity decreased from 7.0 to 3.0%; MOR increased from 8.19 to 12.26 MPa; MOE increased from 1.67 to 2.87 GPa; and compressive strength increased from 16.00 to 27.20 MPa. The thermal properties of the biocomposite also improved as the particle size reduced. The authors also found that the performance of the biocomposite improved significantly with a lower percentage matrix and filler nanoparticle rather than when increasing the percentage of the matrix. The finding of this research also indicates that the properties of the biocomposite can be improved by reducing the particle size of the filler to nanometers without having to increase the adhesive composition.

The findings of another study [52] demonstrated that eco-friendly plywood samples using four various wood species (beech, birch, hornbeam and poplar) bonded with LDPE film of different thicknesses (50, 80, 100 and 150 μm) showed satisfactory physical–mechanical properties. Poplar veneer provided the lowest values for MOR, MOE and TS of all the plywood samples, but the bonding strength was at the same level as birch and hornbeam veneer. Beech plywood samples had the best mechanical properties. An increase in LDPE film thickness improved the physical–mechanical properties of plastic-bonded plywood.

In another paper, low-cost wood–plastic composites (WPCs) without any additives were developed from invasive trees without prior processing and low-grade recycled low-density polyethylene [53]. The authors evaluated different biomass/plastic ratios, particle sizes, and press settings to determine the optimum processing parameters to obtain WPCs with adequate properties. The dimensional stability, WA, MOR, MOE, tensile strength, and tensile moduli were improved at longer press times and higher temperatures for all blending ratios. An increased biomass ratio and particle size were positively correlated with WA and TS and inversely related with MOR, tensile strength, and density due to an incomplete encapsulation of the biomass by the plastic matrix.

In another interesting study, the first attempt to investigate low-density insulation boards made of spruce bark fibers in a wet process was conducted [54]. The insulation boards with densities between 160 and 300 kg/m^3^ were self-bonded. The authors found that widely available bark residues could be successfully utilized as an innovative raw material for efficient eco-friendly thermal insulation products. The thermal conductivity values of the boards were comparable to established insulation boards based on cork or wood fibers. Based on the measured thermal conductivity and zero formaldehyde content, bark fiber insulation panels might be able to compete with conventional insulations if the density can be further reduced, and applications regarding acoustic insulation are also a possibility.

Oil palm trunks (OPT) are considered significant waste products. Usually, the trunks remain on the plantation site for nutrient recycling or burning, which increases insect and fungi populations. This causes environmental problems for the new palm generation or air pollution due to fire. Therefore, the comprehensive review conducted by Nuryawan et al. [55] summarizes the utilization of OPT into products made of oil palm fibers mainly derived from OPT, and its application for the substitution of wood panel products. Some research works have also analyzed oil palm fibers derived from OPT for the exploitation of their potential as raw material to process into various conventional composite panel products, such as plywood and laminated board, particleboard, or binderless and cement board.

Nanocellulose aerogels are a new category of high-efficiency adsorbents for treating oil spills and water pollution. The review provided by Iskandar et al. [56] presents an introduction to nanocellulose-based aerogel and its fabrication approaches. Different applications of nanocellulose aerogel in environmental, medical and industrial fields are presented. Different strategies for the modification of nanocellulose-based aerogel are also critically discussed in this review, presenting the most recent works in terms of enhancing the aerogel performance in oil absorption in addition to the potential of these materials in near future.

In their comprehensive review paper, Ramesh et al. [57] focused on the processing of WPCs along with additives such as wood flour and various properties of WPCs such as mechanical, structural, and morphological properties. Applications of wood-based composites in various sectors such as automotive, marine, defense, and structural applications are also highlighted in this review.

The processing technology, bonding mechanism and performance of thermoplastic-bonded wood-based panels are comprehensively summarized and reviewed in another interesting paper [58]. Meanwhile, the existing problems for this new kind of panel and their future development trends are also highlighted, which can provide the wood industry with foundations and guidelines for using thermoplastics as environmentally friendly adhesives and effectively solving indoor pollution problems.

In recent years, different types of thermoplastic films such as polyethylene, polypropylene, polyvinyl chloride, co-polyamide and co-polyester have been widely used for wood veneer bonding owing to their excellent water resistance, flexibility, easy processing, and secondary melting characteristics. The findings of another study [59] demonstrated that plastic plywood can be produced using an ethylene–vinyl acetate (EVA) film as a wood adhesive via hot press and cold press processes. The results showed that the EVA film featured good gluing ability, and the EVA plywood could be used in indoor environments.

A very promising direction is the use of lignin and its derivatives as an ecological alternative to petroleum-based adhesives. However, being the most common renewable source of phenolic compounds of natural origin, only 1–2% of the huge annual production volume (50–70 million tons) is actually used for the production of value-added products. Lignosulphonates (LS) account for 90% of the total market of commercial lignin. In their review paper, Gonçalves et al. [60] carried out a comprehensive overview of the methods to improve the reactivity of lignin molecules, and techniques to extract, characterize, and improve the reactivity of LS. The most recent advances in the application of LS in wood adhesives with and without their combination with formaldehyde, are also discussed.

The papers from this Special Issue represent only some of the recent developments in eco-friendly wood-based composites. The utilization of recycled plastics, lignin and their derivatives, wood (bark) and agricultural wastes to manufacture wood composites as well as traditional WPCs is highly viable concerning eco-friendliness, and contributes to the improvement of the circular economy. It also saves the usage of virgin materials, thus enhancing sustainability in the production of composite materials. However, most of the proposed methods to manufacture high-performance, eco-friendly wood composites with a lower environmental impact have been studied only in laboratory conditions, can only find use in some nonstructural applications, and have not been introduced in large-scale industrial production as yet. Hence, further research is still needed in order to develop methods for improving reactivity and the selection of suitable crosslinkers for lignin-based adhesives, and modification methods to improve the interfacial adhesion between hydrophilic wood and hydrophobic thermoplastics in order to expand their use in some exterior and structural applications.
